# Ethylene-Vinyl Acetate Copolymers as Potential Thermoplastic Modifiers of Photopolymer Compositions

**DOI:** 10.3390/polym15010131

**Published:** 2022-12-28

**Authors:** Dmitriy A. Bazhanov, Arkadiy A. Poteryaev, Alexey V. Shapagin, Anna A. Shcherbina

**Affiliations:** 1 Frumkin Institute of Physical Chemistry and Electrochemistry (IPCE), Russian Academy of Sciences (RAS), Leninsky Pr. 31-4, 119071 Moscow, Russia; 2 Department of Chemical Technology of Polymer Composite Paint Materials and Coatings, Mendeleev University of Chemical Technology, Miusskaya Sq. 9, 125047 Moscow, Russia

**Keywords:** additive manufacturing, 3D printing, diffusion, phase-state diagram, solubility, thermoplastics, tert-butyl acrylate, vat photopolymerization

## Abstract

The possibility of using thermoplastic polymers in photopolymer compositions for SLA and DLP is discussed in this article. The diffusion and mutual solubility of uncured systems based on tert-butyl acrylate (tBA) and ethylene-vinyl acetate copolymers (EVA) or low-density polyethylene (LDPE) were studied. The solubility and diffusion of tBA with EVA containing 7, 20, and 40 wt.% vinyl acetate (VA) and with LDPE in the temperature range 20–75 °C were studied by optical micro-interferometry method. Phase diagrams of LDPE–tBA, EVA-7–tBA, and EVA-20–tBA systems were obtained. It is shown that the compositions are characterized by the phase-state diagrams of amorphous separation with the upper critical solution temperature (UCST). The concentration dependences of the interdiffusion coefficients as well as dependences of the self-diffusion coefficients on VA content and on temperature were plotted. The activation energy of self-diffusion of EVA and LDPE was calculated. It was shown that the most promising tBA modifier is EVA-40, which is completely soluble at all studied temperature ranges. The obtained data on the mixing of the initial components is valuable for further studies of the processes of structure formation during photocuring of compositions, regulation of the phase structure and, as a consequence, the performance characteristics of the 3D printable materials.

## 1. Introduction

Recently, additive manufacturing (AM) technologies have become popular among research groups working in the field of polymer materials science [1,2,3]. This technology emerged in the 1980s [4,5,6] and was intended for rapid prototyping (RP), but today it is attracting more and more attention in various industries, using additive technologies for 3D printing of structural products [1,4,7]. Due to the wide choice of polymeric materials, additively manufactured products have been used in the automotive [4,8,9], aerospace [4,8,9,10], and architecture/construction industries [11,12,13,14], as well as medicine/health [8,9,15], electronics, [6,11,16,17] and many other fields. Additive technology owes its popularity to the ability to manufacture products with complex geometries oriented toward consumer requirements for individual products [1,4,6,18]. Compared to the molding processes this reduces the financial and time costs of producing of small batch parts [4,18,19,20].

AM allows the formation of three-dimensional objects by adding material layer-by-layer in a ‘bottom-up’ or ‘top-down’ approach [1,2,4,6,7,18,21,22,23,24]. In recent years, among existing AM techniques vat photopolymerization (VP) has become the most widely used [4,25,26]. This technology, in particular, includes stereolithography (SLA) [6,27] and digital light projection (DLP) [9,23,27], which have received the most widespread use. These methods are based on the process of chemical crosslinking (photocuring) of polymeric thermosetting materials using UV laser exposure or UV LEDs [4,6,9,11,28,29,30,31,32]. The main advantages of VP are the high printing resolution (~20 μm) [4,11,26,33,34,35] and the high quality of the resulting surface [26]. Liquid photopolymer resins (photoresins) based on (meth)acrylates capable of free radical polymerization are used as initial materials [2,4,7,11,24,36,37].

It is known that the material obtained by 3D printing has reduced interlayer adhesion and, as a result, products are characterized by low mechanical properties, which limits their application [3,4,6,11,38,39]. In this regard, the addition of modifiers to polymers can improve mechanical, thermal, electrical, and other properties [3,7,11,22]. Thus, to improve the properties of photopolymer compositions, particles of aluminum (III) oxide [40,41] or diamond [42] are used. At the same time, nanomaterials are widely used in vat photopolymerization, such as graphene oxide (GO) nanoparticles [3,31,38], carbon nanotubes (CNT) [23,43,44], titanium (IV) oxide nanoparticles [45,46], and carbon and aramid nanofibers [7].

Analysis of the scientific literature showed that in the field of 3D printing the possibility of using of compatible or limited-compatibility thermoplastic polymers as modifiers of photopolymer compositions has rarely been studied [47,48]. No works were found in which the processes of the interdiffusion of components in the initial uncured systems and during the chemical reaction of in situ photocuring were investigated. Thus, an important problem in the modification of photopolymer resins is the limited choice of compatible polymer components and the lack of information on phase equilibria in their mixtures with modifiers.

Obtaining of the information about the solubility of components and the kinetics of diffusion processes in the systems of photopolymer resins modified with thermoplastics at different stages of the curing process is fundamental for predicting the phase structure of the cured composition [49,50,51,52,53] and, consequently, obtaining a set of given performance characteristics of the material.

Thus, the goal of this work was a comprehensive study of the phase equilibria and interdiffusion of components of initial photopolymer compositions based on tert-butyl acrylate (tBA) modified with ethylene-vinyl acetate copolymers (EVA) and low-density polyethylene (LDPE) to form the physico-chemical basis for the preparation of cured heterogeneous photocompositions characterized by increased physical and mechanical properties, including at reduced temperatures.

## 2. Materials and Methods

### 2.1. Materials

The photocurable monomer tBA and thermoplastic polymers LDPE and EVA, containing 7 wt.%, 20 wt.%, and 40 wt.% of VA, respectively, were used.

The tBA and thermoplastic polymers were characterized by DSC, MALDI, and refractometry methods. VA content of EVA (*C_VA_*), glass transition temperatures (*T_g_*), melting temperatures (*T_m_*), densities (*ρ*), refractive indexes (*n_D_*^20^), and molecular weights (*M_w_*) are provided in Table 1.

### 2.2. Sample Preparation

Films of EVA and LDPE were prepared by pressing on a hydraulic press at temperatures above their *T_m_*. Pressing temperatures were: 185 °C for LDPE; 120 °C for EVA-7; 100 °C for EVA-20 and 90 °C for EVA-40. The thickness of the films was 100–120 µm.

### 2.3. Methods Characterization

#### 2.3.1. Refractometry

To interpret the results obtained by optical micro-interferometry, for compositions with limited compatibility, information on the refractive indexes [54] of the components at the corresponding temperatures was required.

The difference in refractive indexes of the components of the diffusion system determines the total number of interference bands (N) and the increment of refractive index and concentration per fringe (Δn), Equation (1):

(1) $$N=\frac{n_{1}-n_{2}}{∆n}\;$$

For the wedge angle *θ* = 2° the parameter Δn was 0.003 [55]. This was necessary for determination of the compositions of coexisting phases in the mixtures with amorphous phase separation.

Temperature dependences of refractive indexes of studied objects were obtained on an Abbe refractometer ATAGO NAR-2T (Atago Co., Ltd., Tokyo, Japan) in the ‘heating–cooling’ mode in the temperature range 20–120 °C with an accuracy of ±0.0001. The values were chosen to cover the range of diffusion measurements and possible phase transitions of the components. Step of measurements was 5–10°, temperature control time was at least 15 min at each stage.

#### 2.3.2. Optical Micro-Interferometry

The diffusion and mutual solubility of the components of the tBA–thermoplastic polymer systems were studied by the optical micro-interferometry method [55,56]. This method is based on the phenomenon of multibeam interference between two semipermeable glass surfaces with a small wedge angle (*θ* ≤ 2°). Due to the difference in the path of the transmitted and reflected rays of monochromatic light, an interference pattern emerges. It is localized on the lower surface of the wedge and is composed of alternating light and dark fringes. The curvature of interference isoconcentration fringes and the appearance of the phase boundary or phase particles in the diffusion zone provide information about the nature of changes in the refractive indexes of the solution in the diffusion zone and, consequently, about the mutual solubility of the components. The kinetics of motion of isoconcentration interference fringes were used to calculate the mutual diffusion coefficients.

An optical diffusiometer ODA-2 IPCE (IPCE RAS, Moscow, Russia) with a helium-neon laser (*λ* = 632.8 nm) was used for measurements [55]. The temperature control system with a HANYOUNG NX2 temperature controller (Hanyong Nux Co., Ltd., Incheon, Korea) was used to maintain a constant temperature in the interference cell within the range of 20–75 °C with an accuracy of ±0.5°. The inner surfaces of the glasses were coated with a thin translucent layer of Ni+Cr alloy.

A 3 × 4 mm sample of EVA or LDPE film was placed between two glasses of a diffusion cell forming a wedge angle *θ*~2° between them. The interference fringes were oriented parallel to the wedge edge and perpendicular to the diffusion front. The assembled cell was placed in a diffusiometer cell, thermostatted to the temperature of the experiment, and the monomer tBA was injected into the wedge gap. The moment of contact of the fronts was observed on the monitor screen at magnification of the optical microscope and was considered to be the beginning of the diffusion mixing of the components. Interferograms of the interdiffusion zones were recorded periodically with an interval of 1–10 min, depending on the diffusion rate. The interdiffusion coefficients were measured in isothermal mode. Compositions of coexisting phases for plotting phase-state diagrams were determined in the heating–cooling mode with a step of 20° and holding at each step until equilibrium state was reached.

**Figure 1:**
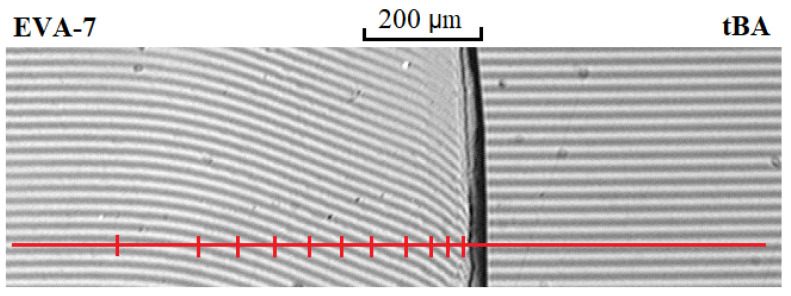
Interferogram of the interdiffusion zones of EVA-7–tBA at temperature 75 °C and diffusion time 16 min. Explanation of the figure in the text.

The previously described method [57] was used to construct phase diagrams. In the interdiffusion zone, a solid line parallel to the interference fringes in the pure component regions was drawn (Figure 1, red line). Then, the number of intersections of this line with the curved lines of the interference pattern to the left and to the right of the phase boundary was calculated. The obtained values were multiplied by C = 1/N (N is calculated according to Equation (1); C–the change in concentration between the nearest intersections) and were determined the compositions of the coexisting phases on phase diagram.

## 3. Results and Discussion

### 3.1. Refractometry Results

**Figure 2:**
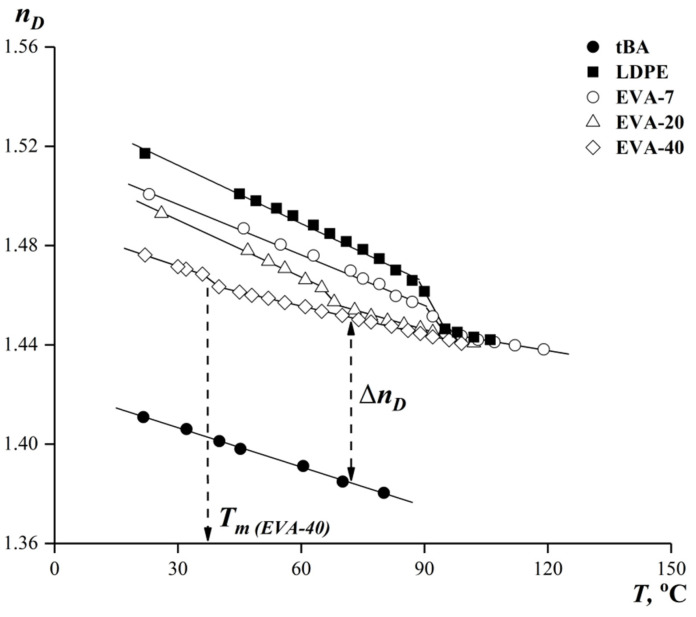
Temperature dependences of the refractive index of tBA, LDPE, EVA-7, EVA-20, EVA-40.

Figure 2 shows the temperature dependences of the refractive indexes of the studied objects obtained in the above-described modes.

It can be seen that, within one phase, state the temperature dependences were linear for all the studied components. Steps on the dependances for EVA and LDPE correspond to their melting temperatures *T_m_*. Using Equation (1) for obtained data, the total number of interference bands for each composition was calculated.

### 3.2. Diffusion Zones

**Figure 3:**
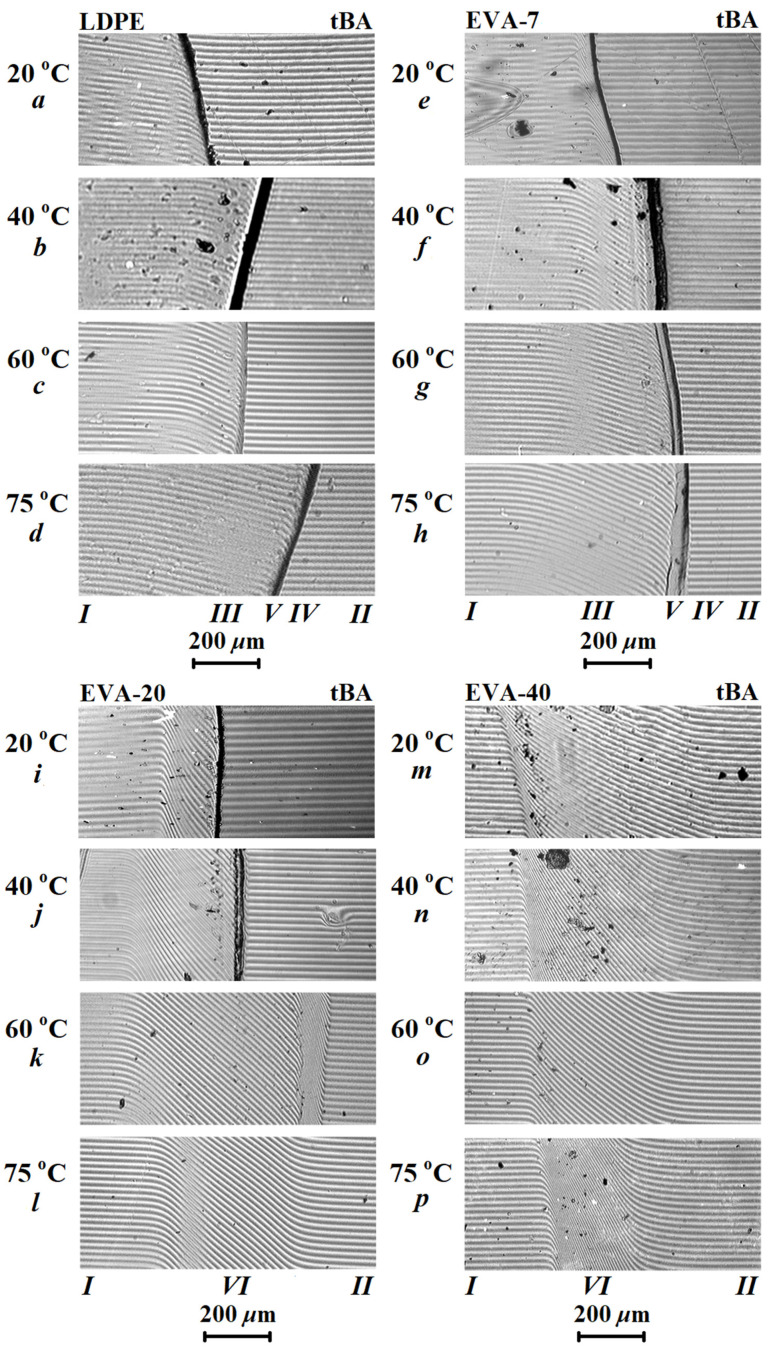
The effect of temperature and VA content in the copolymer on the character of interferograms in the tBA-modifier systems. VA concentrations: 0 wt.% (**a**–**d**), 7 wt.% (**e**–**h**), 20 wt.% (**i**–**l**), and 40 wt.% (**m**–**p**). The temperature is shown near the interferograms. Diffusion times range from 2 to 16 min, depending on temperature and composition.

Figure 3 shows typical interferograms of diffusion zones spontaneously formed after conjugation of components in the temperature range 20–75 °C. The upper temperature of the experiment was limited by the high volatility of tBA, which makes it impossible to obtain reliable results at higher temperatures.

It can be seen that there was either a smooth curvature of the interference fringes as a result of a smooth change in the refractive index, or a phase boundary appeared in the interdiffusion zone. The phase boundary separated the region of dissolution of tBA in thermoplastic from the region of dissolution of thermoplastic in tBA. Thus, the interference patterns (Figure 3) indicate the areas of: pure LDPE or EVA (I) and tBA (II) components, region of solution of tBA in thermoplastic modifier (III), region of dissolution LDPE or EVA in tBA (IV), phase boundary (V), and interdiffusion region (VI) for fully compatible systems.

**Figure 4:**
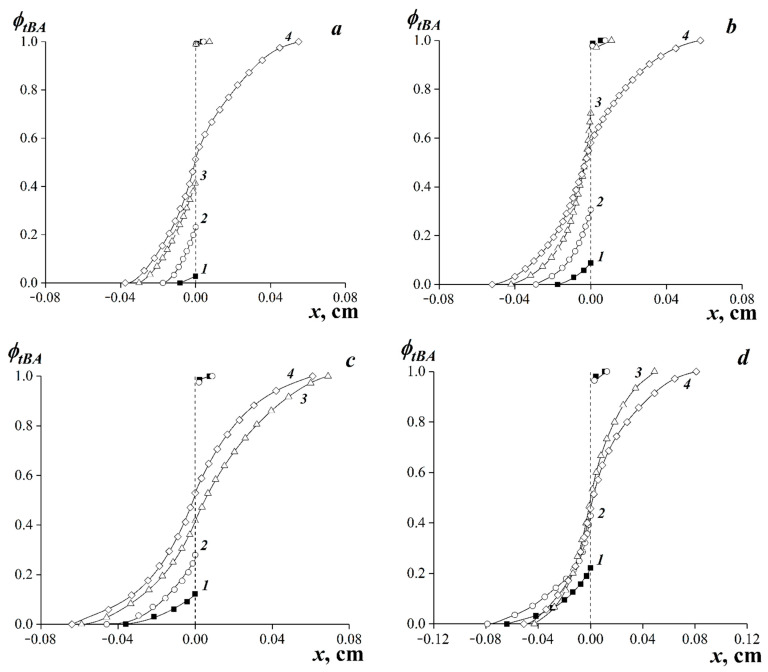
Concentration distribution profiles of the interdiffusion zones for the systems: LDPE–tBA (**1**), EVA-7–tBA (**2**), EVA-20–tBA (**3**), and EVA-40–tBA (**4**). Temperatures: 20 °C (**a**), 40 °C (**b**), 60 °C (**c**), and 75 °C (**d**). Time from the beginning of the experiment: **1a**–**4a**—16 min; **1b**–**4b**—16 min; **1c**–**3c**—16 min; **4c**—4 min; **1d**, **2d**—16 min; **3d**, **4d**—2 min.

Using the obtained interferograms (Figure 3), concentration profiles characterizing the distribution of tBA in the interdiffusion zone were plotted (Figure 4). In the case of limited component compatibility, the profiles were plotted relative to the phase boundary.

It was found that the EVA-40–tBA system (Figure 3m–p) was fully compatible over the entire temperature range. In the interdiffusion zone, a continuous concentration distribution profile was formed during the transition from EVA-40 to tBA, and the concentration distribution curve was practically symmetric about the middle of the interdiffusion zone (Figure 4a–d, curve 4). This probably can be explained due to the proximity of the solubility parameters of the components of this composition.

Reducing the VA content of the sample resulted in partial compatibility at low temperatures for tBA with EVA-20 and at all temperature ranges for EVA-7–tBA and LDPE–tBA systems. At the same time, for EVA-7–tBA and LDPE–tBA systems, there were mostly no signs of dissolution of thermoplastic in the monomer tBA on interferograms.

### 3.3. Phase State Diagrams in the tBA-Based Compositions

**Figure 5:**
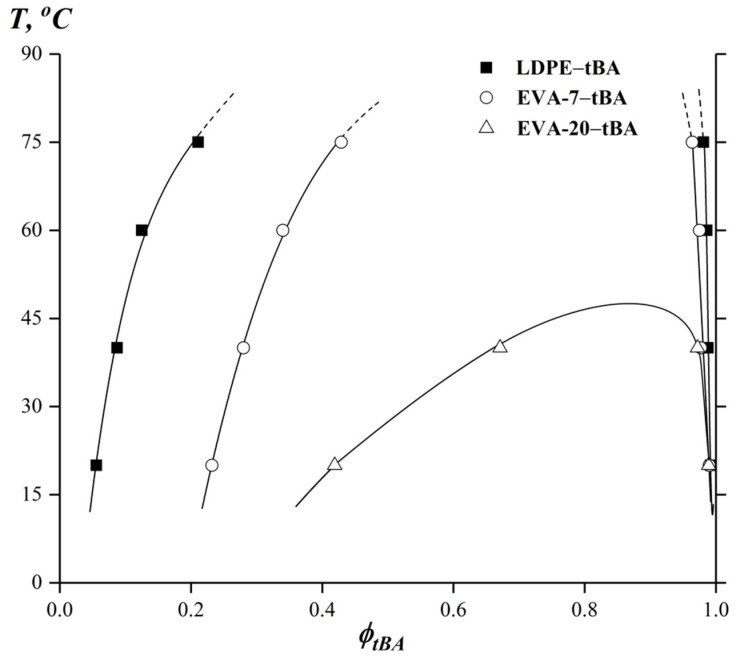
Binodal curves for tBA systems with LDPE, EVA-7, and EVA-20.

Using obtained concentration profiles (Figure 4) phase diagrams for LDPE–tBA, EVA-7–tBA and EVA-20–tBA systems were constructed (Figure 5).

It was found that the solubility of the components improved with increasing temperature. The compositions are characterized by a phase diagram of amorphous phase separation with the UCST [58,59]. For systems with LDPE and EVA-7 the critical temperature is located in the region of high-volatility tBA and was not experimentally determined (Figure 5).

A Flory–Huggins theory of polymer solutions was used to construct the dome of binodal curves and calculate spinodal curves [59,60,61]. The critical temperatures of the systems were calculated using the temperature dependences of the Flory–Huggins interaction parameter *χ*, Equation (2) [59,60]:

(2) $$\chi =\frac{\frac{ln\left(\varphi_{1}^{\prime \prime }/\varphi_{1}^{\prime }\right)}{r_{1}}-\frac{ln\left(\varphi_{2}^{\prime \prime }/\varphi_{2}^{\prime }\right)}{r_{2}}}{2\left(\varphi_{2}^{\prime }-\varphi_{2}^{\prime \prime }\right)},\;$$

where *φ*_1_*′*, *φ*_2_*′*, *φ*_1_*″*, and *φ*_2_*″* are compositions of coexisting phases on the left and right branches of the binodal curve, respectively; *r*_1_, *r*_2_ are degrees of polymerization of components.

**Figure 6:**
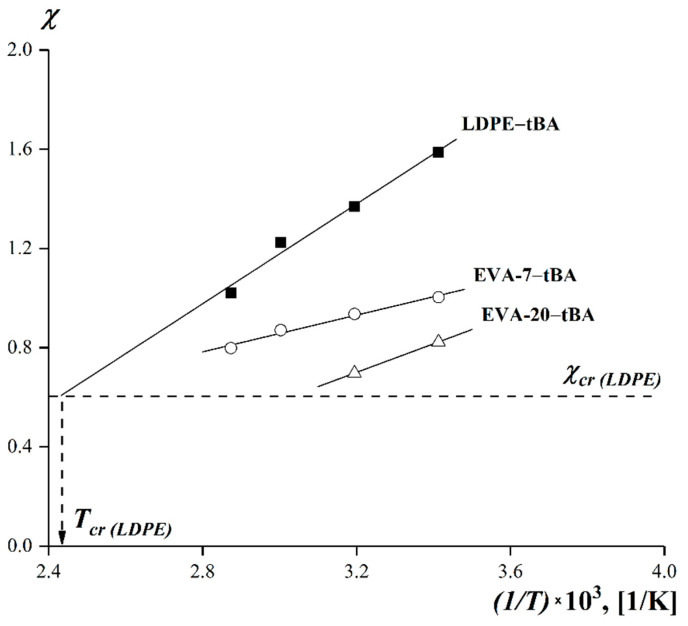
Temperature dependence of the Flory–Huggins parameter when mixing tBA with LDPE, EVA-7, and EVA-20.

It is shown that the temperature dependences (Figure 6) of the Flory–Huggins interaction parameter for systems with limited compatibility are linear in *χ*—(*1*/*T*) coordinates.

The critical temperatures *T_cr_* were determined by extrapolating *χ*—(*1*/*T*) dependences to the intersection with the critical value of the Flory–Huggins parameter *χ_cr_*. The *χ_cr_* parameters and the critical concentrations *φ_cr_* were calculated using the following Equations (3) and (4) [59,60]:

(3) $$\chi_{cr}=\frac{1}{2}{\left(\frac{1}{\sqrt{r_{1}}}+\frac{1}{\sqrt{r_{2}}}\right)}^{2}$$

(4) $$\varphi_{cr}=\frac{\sqrt{r_{1}}}{\sqrt{r_{1}}+\sqrt{r_{2}}}\;$$

Spinodal curves separating the regions of metastable and labile solutions were calculated using Equations (5), (6a), and (6b) from the Flory–Huggins theory, using software [62] developed in the Structural and Morphological Research Laboratory, IPCE RAS:

(5) $$\frac{1}{r_{1}\varphi_{1,s}}+\frac{1}{r_{2}\varphi_{2,s}}-2\chi =0.\;$$

(6a) $$\varphi_{1,s}=\frac{-\left(r_{1}-r_{2}-2\chi r_{1}r_{2}\right)+\sqrt{{\left(r_{1}-r_{2}-2\chi r_{1}r_{2}\right)}^{2}-8\chi r_{1}r_{2}{}^{2}}}{4\chi r_{1}r_{2}}.\;\;$$

(6b) $$\varphi_{2,s}=\frac{-\left(r_{1}-r_{2}-2\chi r_{1}r_{2}\right)-\sqrt{{\left(r_{1}-r_{2}-2\chi r_{1}r_{2}\right)}^{2}-8\chi r_{1}r_{2}{}^{2}}}{4\chi r_{1}r_{2}}.\;\;$$

Table 2 summarizes the critical values of the Flory–Huggins parameters, critical temperatures, and concentrations.

**Figure 7:**
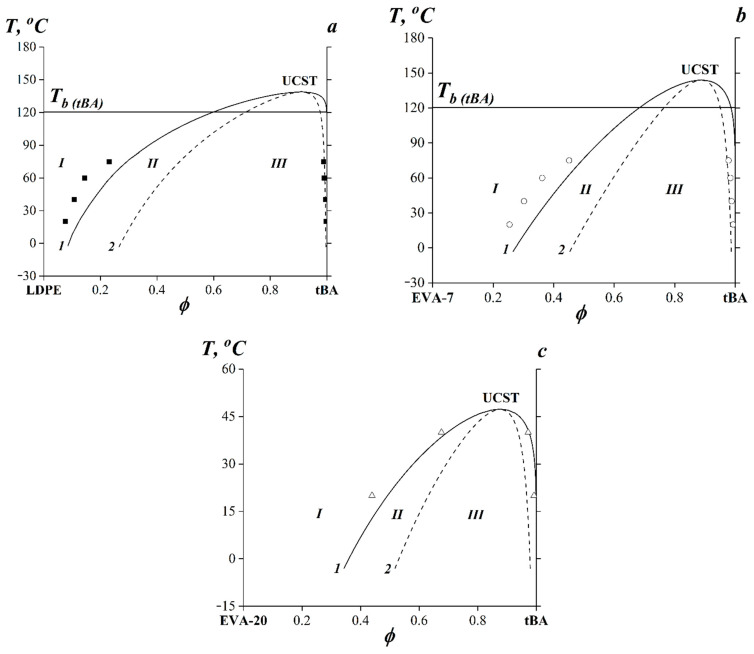
Phase-state diagrams for LDPE–tBA (**a**), EVA-7–tBA (**b**), EVA-20–tBA (**c**) systems. The markers indicate the experimental data. Curve **1** (black solid line)—calculated binodal curves. Curve **2** (black dashed line)—calculated spinodal curves. **I**—the region of true solutions, **II**—the region of metastable states, **III**—the region of labile structures.

Figure 7 shows phase-state diagrams for LDPE–tBA, EVA-7–tBA and EVA-20–tBA systems. Experimental and calculated data are shown.

It can be seen that the calculated UCTS values for the systems LDPE–tBA and EVA-7–tBA are above the boiling point *T_b_* of tBA (Figure 7a,b), which is 121 °C. It should be noted that the binodal curves of all systems were asymmetric and, in accordance with the terms of the Flory–Huggins theory, the critical concentrations were shifted toward the component with the lower *M_w_* (i.e., tBA).

It should be noted that, with an increase in VA content in the copolymer, the UCST decreased, the heterogeneous region became less extended, and the critical point slightly shifted to the middle region of compositions. The position of the right branch of the binodal curve (polymer solubility in tBA) on the temperature concentration field of the phase diagram remained practically unchanged. According to the position of the left branch of the binodal, the solubility of tBA in the modifier increased sharply with VA content from 0 (LDPE) to 20 wt.% (EVA-20) augmentation.

### 3.4. Diffusion in tBA-(LDPE or EVA) Systems

**Figure 8:**
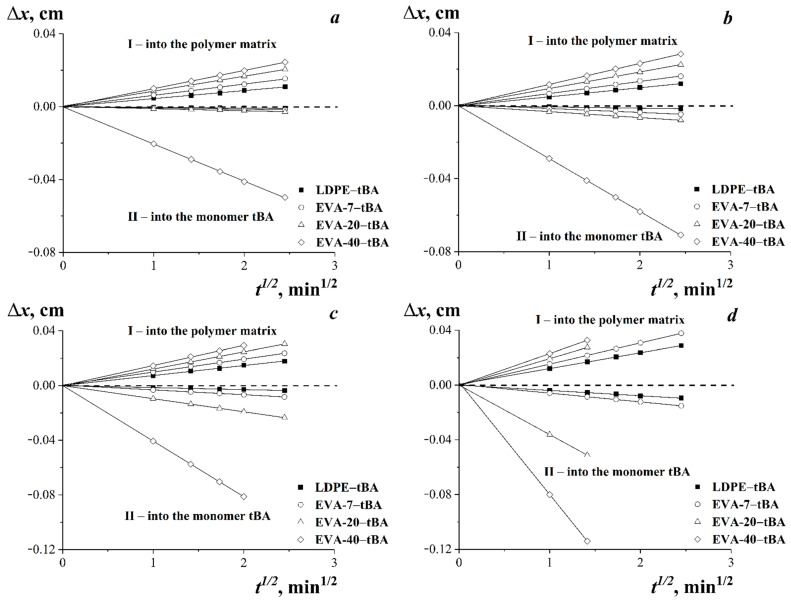
Kinetics of motion of isoconcentration planes of LDPE–tBA, EVA-7–tBA, EVA-20–tBA, and EVA-40–tBA systems. Temperatures: 20 °C (**a**), 40 °C (**b**), 60 °C (**c**), and 75 °C (**d**).

The kinetics of movement of diffusion fronts of tBA in EVA or LDPE (*I*) and EVA or LDPE in tBA (*II*) in the diffusion coordinates Δ*x*—*t*^1/2^ (Δ*x* is the penetration depth of the diffusing component; *t* is the observation time) are shown in Figure 8.

It is shown that the sizes of diffusion zones increased in time. All dependences in the coordinates Δ*x*—*t*^1/2^ are linear, indicating the diffusion mechanism of component mixing in the LDPE–tBA and EVA–tBA systems in the region of true solutions. The character of the dependences did not change when the temperature increased (Figure 8).

The limiting partial diffusion coefficients *D_V_* in LDPE–tBA and EVA–tBA systems were calculated from the obtained dependences using the following expression from Fick’s second law Equation (7), [58]:

(7) $$D_{V}=\frac{1}{2}{\left(\frac{∆x}{t^{1/2}}\right)}^{2}$$

The Matano–Boltzmann analysis was used to obtain the values of interdiffusion coefficients of components in the entire concentration range in the region of true solutions. The interdiffusion coefficients were calculated according to the following Equation (8) [58,63]:

(8) $$D_{V}\left(c_{i}\right)=-\frac{1}{2t}\cdot {{\left(\frac{\partial c}{\partial x}\right)}^{-1}|}_{x_{i}}\cdot \overset{c_{i}}{\underset{c_{0}}{\int }}\left(x_{M}-x_{i}\right)dc,$$

where *t* is the diffusion time; *c*_0_ is the minimum concentration of the substance under study; *c_i_* is the concentration at the point with *x_i_* coordinate; and *x_M_* is the coordinate of the Matano plane.

**Figure 9:**
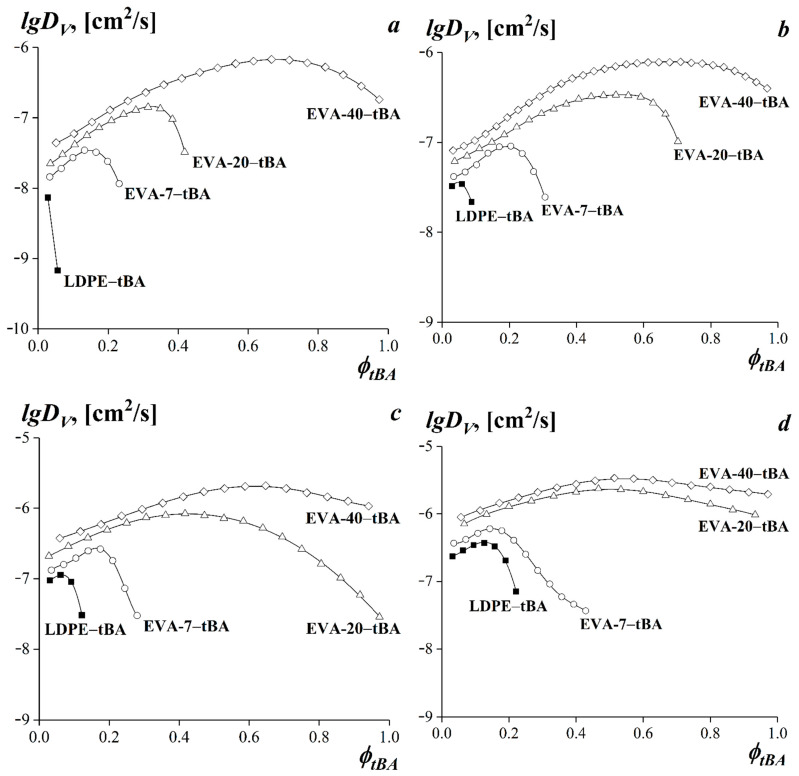
Concentration dependences of interdiffusion coefficients of LDPE–tBA, EVA-7–tBA, EVA-20–tBA, and EVA-40–tBA systems at 20 °C (**a**), 40 °C (**b**), 60 °C (**c**), and 75 °C (**d**).

The concentration dependences of the interdiffusion coefficients of the studied compositions are shown in Figure 9. For the systems with limited compatibility, the dependences in the coordinates *lgD_V_*—*φ_tBA_* were determined only in the region of true solutions.

It is shown that the obtained concentration dependences of interdiffusion coefficients are characterized by curves with a maximum of the diffusion coefficient. An increase in temperature leads to an increase in the values of the interdiffusion coefficients in the entire concentration range.

As the system composition approaches the binodal curve, a decrease in the interdiffusion coefficient is observed (Figure 9a–d). We associate the decrease in the value of the interdiffusion coefficient in the region of true solutions with the value of the thermodynamic correction (*∂μ*_1_/*∂φ*_1_) [59,61], which tends toward zero when composition is close to the labile region (to the spinodal), Equation (9):

(9) $$D_{V}\approx D_{1}^{*}{\left(\frac{\partial \mu_{1}}{\partial \varphi_{1}}\right)|}_{T,P}$$

where D*_1_ is the partial mobility of the solvent in solution, *μ*_1_ is the chemical potential of the solvent, and *φ*_1_ is the volume fraction of the solvent.

### 3.5. Dependences of the Limiting Values of Interdiffusion Coefficients on the Experimental Temperature and on the VA Content in the Copolymer

It is known that the limiting value of interdiffusion coefficient at solvent concentration tending toward zero characterizes the mobility of solvent molecules in the polymer matrix (phase) [59,64]. In this case, it can be taken as the polymer self-diffusion coefficient *D*_2_ [61]. Information on this value and its temperature and concentration dependances is of fundamental importance for analysis of the mechanism of translational mobility of individual macromolecules in various solvents [57,59,61].

The limiting values of the interdiffusion coefficients can be obtained by extrapolation of the dependence in coordinates *lgD_V_*—*φ* to the region of infinitely dilute solutions [58]. The values of self-diffusion coefficients *D*_2_ for LDPE and EVA obtained in the temperature range 20–75 °C are given in Table 3.

**Figure 10:**
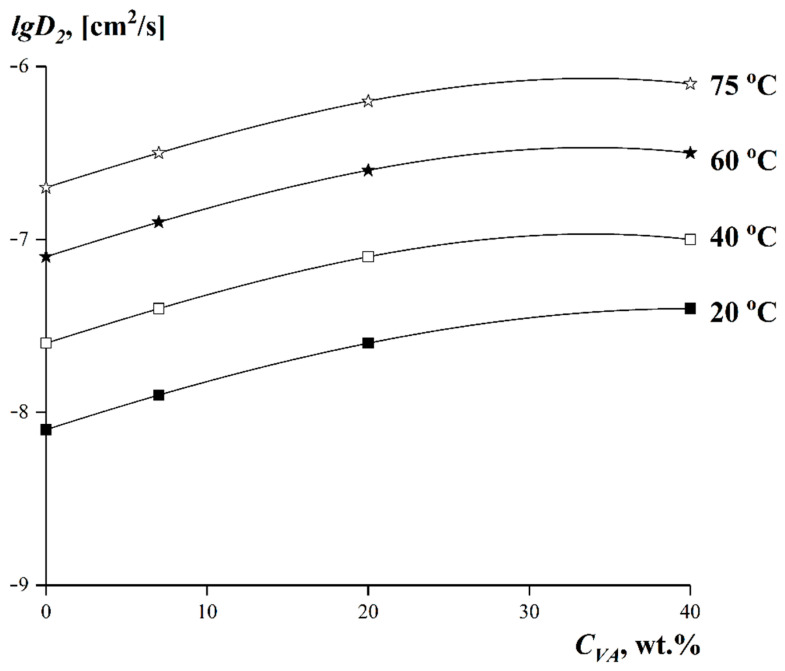
Dependences of the limiting interdiffusion coefficient at *φ_tBA_ →* 0 on the VA content in EVA. Temperatures are indicated in the figure.

Figure 10 shows the dependences of self-diffusion coefficients *D*_2_ on copolymer composition at different experimental temperatures.

**Figure 11:**
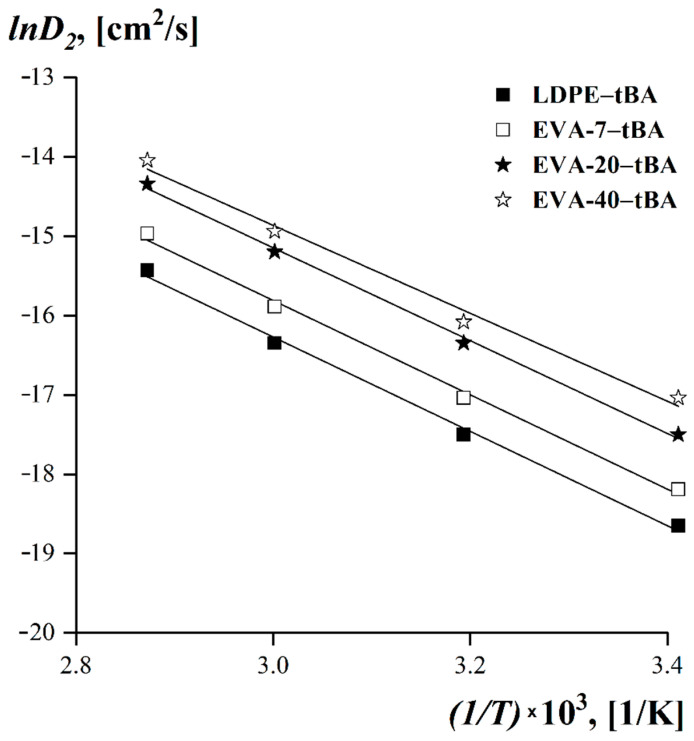
Temperature dependences of the limiting interdiffusion coefficients at *φ_tBA_ →* 0 for LDPE– tBA, EVA-7–tBA, EVA-20–tBA, and EVA-40–tBA systems.

It can be seen that over the whole temperature range covered by our experiments, the values of the EVA self-diffusion coefficients grew smoothly as the VA content in the copolymer increased. The temperature dependences of the self-diffusion coefficients at *φ_tBA_ →* 0 in the Arrhenius equation coordinates are shown in Figure 11.

The temperature dependences of the self-diffusion coefficients in Arrhenius coordinates were linear for all investigated systems. Using the presented data (Figure 11) the self-diffusion activation energy of LDPE and EVA was calculated from Arrhenius equation for the dependence of the diffusion coefficient on temperature, Equation (10) [61]:

(10) $$D_{V}=D_{o}\cdot exp\left(-\frac{E_{a}}{RT}\right),$$

where *D*_0_ is the constant that depends on the properties of the substance and the diffusion mechanism, *E_a_* is the activation energy of diffusion, *R* is the universal gas constant, and *T* is the absolute temperature.

The calculated data is shown in Table 4.

It was found that with increasing VA content in the EVA copolymer the self-diffusion activation energy decreased, which, consequently, was accompanied by an increase in the diffusion rate of tBA molecules into the thermoplastic matrix and improvement of the components’ mutual solubility.

## 4. Conclusions

Modification of the photopolymer compositions by introducing compatible and partially compatible thermoplastics has significant advantages compared to the insoluble modifiers (carbon nanotubes or metal oxide particles), since no special techniques of modifier introduction and particle agglomeration prevention are required. At the same time, the phase structure of the modified composition formed in the photocuring process determines the set of performance characteristics of the 3D material. In order to predict the type of final-phase structure and its properties and characteristics, information about the phase equilibria and diffusion process parameters of initial components of the photopolymer compositions is necessary.

The obtained results show that the high values of interdiffusion coefficients (10^−6^–10^−7^ cm^2^/s) and full compatibility of the components of EVA-40–tBA systems even at room temperature and make EVA-40 copolymer the most promising thermoplastic modifier for tBA.

The effect of VA content in EVA copolymer on the phase diagrams, limiting diffusion coefficients, and self-diffusion activation energy was analyzed. The effect of temperature and tBA content on the mixing kinetics is shown. Approaches following from the Flory–Huggins theory [59,60,61] were applied to obtain experimentally inaccessible points of the phase diagrams.

The obtained information is not only of applied but also of fundamental significance. In continuation of this work, the structure formation during photocuring of compositions will be investigated. The data obtained in the current investigation results on phase equilibria and mixing kinetics at different the VA content in EVA–tBA compositions will be used to regulate the phase structure and, as a consequence, the performance characteristics of 3D printable materials.

## Figures and Tables

**Table 1:** Properties of the research objects.

Object	Producer	*C_VA_*, [wt. %]	*T_g_*, [°C]	*T_m_*, [°C]	*ρ*, [g/cm^3^]	*n_D_* ^20^	*M_w_*, [Da]
tBA	Acros Organics BVBA, Geel, Belgium	—	—	−69.0 ^2^	0.883 ^2^	1.4119 ^3^	128
LDPE	Naftan, New Polotsk, Belarus	0	−20.1 ^1^	113.3 ^1^	0.916 ^2^	1.5193 ^3^	2900 ^4^
EVA-7	Savilen, Kazan, Russia	5.0–7.0 ^2^	−34.3 ^1^	95.4 ^1^	0.925 ^2^	1.5026 ^3^	2800 ^4^
EVA-20	Total Fina Elf S.A., Kulbua, France	19.0–21.0 ^2^	−34.6 ^1^	53.3 ^1^	0.940 ^2^	1.4979 ^3^	2500 ^4^
EVA-40	DuPont de Nemours, Wilmington, DE, USA	40.0 ^2^	−30.6 ^1^	49.3 ^1^	0.966 ^2^	1.4775 ^3^	2000 ^4^

^1^ obtained using DSC—NETZCH DSC 204 F1 Phoenix (Netzsch-Gerätebau GmbH, Selb, Germany). ^2^ provided by the manufacturer. ^3^ according to refractometry data at 20 °C and *λ* = 589.3 nm—ATAGO NAR-2T (Atago Co. Ltd., Tokyo, Japan). ^4^ obtained using MALDI-TOF/TOF UltraFlex II (Bruker Daltonics, Bremen, Germany).

**Table 2:** The critical values for LDPE–tBA, EVA-7–tBA, and EVA-20–tBA systems according to the Flory–Huggins theory.

System	*χ_cr_*	*T_cr_*, [°C]	*φ_cr_*
LDPE–tBA	0.6035	138	0.9102
EVA-7–tBA	0.6345	143	0.8877
EVA-20–tBA	0.6514	47	0.8761

**Table 3:** Limiting values of the interdiffusion coefficients for infinitely dilute tBA solutions (*φ_tBA_ →* 0) at the different experimental temperatures.

System	*T_exp_*, [°C]	*lgD*_2_, [cm^2^/s]	System	*T_exp_*, [°C]	*lgD*_2_, [cm^2^/s]
LDPE–tBA	20	−8.1	EVA−20–tBA	20	−7.6
	40	−7.6		40	−7.1
	60	−7.1		60	−6.6
	75	−6.7		75	−6.2
EVA−7–tBA	20	−7.9	EVA−40–tBA	20	−7.4
	40	−7.4		40	−7.1
	60	−6.9		60	−6.5
	75	−6.5		75	−6.1

**Table 4:** Self-diffusion activation energy at *φ_tBA_ →*0 for LDPE–tBA and EVA–tBA systems.

System	*E_a_*, [kJ/mol]
LDPE–tBA	49.37
EVA-7–tBA	49.36
EVA-20–tBA	48.28
EVA-40–tBA	46.83

## Data Availability

The data presented in this study are available on request from the corresponding author.
